# How does servant leadership affect the job satisfaction of tourism employees? The mediating role of psychological capital

**DOI:** 10.3389/fpsyg.2026.1956847

**Published:** 2026-10-07

**Authors:** Ziwei Zhu

**Affiliations:** School of Business, Anhui Xinhua University, Hefei, China

**Keywords:** emotional labor, employee perception, job satisfaction, psychological capital, servant leadership, tourism industry

## Abstract

**Introduction:**

In the service-intensive tourism industry, employee wellbeing is a critical determinant of service quality and organizational performance. Drawing on Positive Organizational Behavior and Conservation of Resources (COR) theory, this study examines the mechanism linking perceived servant leadership to job satisfaction among tourism employees, specifically investigating the mediating role of psychological capital.

**Design/Methodology/Approach:**

Empirical data were collected via an anonymous questionnaire survey of 200 frontline employees from tourism enterprises (travel agencies, scenic spots, and hotels). Hierarchical regression analysis and Bootstrap mediation analysis were employed to test the hypotheses.

**Findings:**

The results indicate that: (1) Perceived servant leadership is significantly positively associated with job satisfaction (*p* < 0.001); (2) Employee-perceived servant leadership is significantly positively associated with employees’ psychological capital; and (3) Psychological capital partially mediates the relationship between employee-perceived servant leadership and job satisfaction.

**Originality/Value:**

These findings extend the theoretical understanding of leadership and wellbeing in the tourism context and offer practical insights for fostering a sustainable workforce through leadership development.

## 1 Introduction

As a vital component of modern service industries, tourism is characterized by its heavy reliance on interpersonal interactions and emotional labor. Tourism employees must not only fulfill basic service tasks but also continuously regulate their emotions to meet tourists’ service expectations. However, prolonged high-intensity emotional labor, irregular working hours, and excessive workloads can lead to accelerated depletion of employees’ psychological resources, thereby affecting their job satisfaction and organizational commitment. Research indicates that job satisfaction not only impacts individuals’ physical and mental health but also directly influences service quality, customer satisfaction, and corporate performance. Therefore, investigating the formation mechanisms of job satisfaction in tourism contexts holds significant theoretical and practical implications.

In organizational management research, leadership behavior is widely recognized as a critical contextual factor influencing employee attitudes and behaviors. Servant leadership—rooted in Greenleaf’s (1970) servant leadership philosophy of “serving employees and empowering them”—has gained academic attention for its emphasis on fostering employee growth through care, empowerment, and moral exemplification (Liden et al., 2008; Lan et al., 2020). Existing research has shown that servant leadership is positively associated with job satisfaction, organizational commitment, and work engagement (e.g., Zhang and Liu, 2020). However, most current studies analyze leadership behavior from objective characteristics or managerial perspectives, largely overlooking employees’ subjective perceptions. In reality, leadership behavior can only be translated into employees’ psychological responses and behavioral outcomes when it is perceived and internalized by them. This perceptual perspective, although theoretically important, remains underexplored in the tourism literature—constituting the first research gap (Gap 1) that this study addresses.

Furthermore, the psychological mechanisms through which servant leadership influences employees’ job satisfaction require further exploration. While some studies suggest that positive leadership behaviors can foster employees’ wellbeing (Cao et al., 2019); Dai et al., 2021), most research remains at the outcome level, lacking detailed explanations of the underlying mechanisms—this constitutes the second research gap (Gap 2). Psychological capital (PsyCap), a developable positive psychological resource encompassing hope, self-efficacy, resilience, and optimism (Luthans et al., 2007), is recognized as a crucial foundation for individuals to cope with work stress and maintain positive mental states (Avey et al., 2010). In high-pressure, emotionally demanding tourism environments, PsyCap may serve as a pivotal bridge between leadership behaviors and employee wellbeing; yet empirical evidence on this mediating mechanism in the tourism context is scarce—the third research gap (Gap 3).

Based on the aforementioned analysis, this study addresses the three research gaps identified above by focusing on frontline tourism employees. Specifically, it pursues three objectives: (1) to examine the relationship between employee-perceived servant leadership and job satisfaction (addressing Gap 1); (2) to investigate whether psychological capital mediates this relationship (addressing Gap 2); and (3) to test the proposed model in the tourism industry, a high-emotional-labor context in which the mechanism has rarely been examined (addressing Gap 3).

From the perspective of employee perception, the study constructs and tests a “servant leadership—psychological capital—job satisfaction” mechanism model. In doing so, it contributes to the tourism and organizational behavior literatures in three ways: it extends servant leadership research by shifting the analytical focus from leaders’ objective behaviors to employees’ subjective perceptions; it identifies PsyCap as an internal resource-transmission mechanism linking perceived leadership to job satisfaction; and it provides contextual evidence from a service-intensive, emotionally demanding industry. The remainder of the paper is organized as follows: Section 2 reviews the literature and develops the hypotheses; Section 3 describes the methods; Section 4 presents the empirical results; and Section 5 discusses the findings and their implications.

## 2 Literature review and hypotheses

To anchor the hypotheses, this study draws on two complementary theoretical perspectives. First, positive organizational behavior (POB) focuses on state-like, developable psychological capacities that can be measured, developed, and managed for performance improvement (Luthans et al., 2007); PsyCap—comprising hope, self-efficacy, resilience, and optimism—is its core construct. Second, conservation of resources (COR) theory posits that individuals strive to obtain, retain, and protect valued resources, and that resource-rich individuals are better able to cope with demands and to accumulate further resources (Hobfoll, 1989). Together, these perspectives suggest that supportive leadership acts as a situational (external) resource that enables employees to build personal (internal) resources such as PsyCap, which in turn promote positive work attitudes such as job satisfaction. This resource-based logic underlies each of the hypotheses developed below.

### 2.1 Employee-perceived servant leadership

Servant leadership was first proposed by Greenleaf (1970) under the label of servant leadership, which centers on prioritizing the service of employees as the leader’s foremost mission, with organizational goals being achieved through addressing staff needs. Unlike traditional performance- or authority-driven approaches, this model emphasizes care, active listening, empowerment, and employee development (Liden et al., 2008). In this study, the construct is conceptualized in line with Greenleaf’s (1970) servant leadership and is measured from the employees’ perspective using the Servant Leadership Scale developed by Liden et al. (2008). It should be noted that earlier Chinese versions of this manuscript used "service-oriented leadership" as a translation of servant leadership; to align with the international literature, the present manuscript uniformly adopts the term servant leadership. This terminological clarification is important because the study examines how employees perceive leaders’ servant behaviors rather than how leaders describe their own behavior. Indeed, research has shown that employees’ subjective perceptions of leadership behaviors predict their attitudes and outcomes more accurately than leaders’ self-assessments or objective actions. Employee-perceived servant leadership therefore refers to the comprehensive evaluation of leaders’ servant behaviors based on employees’ personal work experiences.Research indicates that servant leadership fosters supportive work environments, enhancing employees’ sense of respect and self-worth and thereby elevating their positive emotional experiences (Lan et al., 2020; Zhang et al., 2021). Job satisfaction, as a comprehensive positive evaluation of work conditions, is significantly influenced by leaders’ caring and supportive behaviors. From a COR perspective, leaders’ care and resource support help employees conserve psychological resources that would otherwise be depleted by emotional labor, and resource availability is in turn associated with more positive evaluations of the work situation (Hobfoll, 1989). In the tourism industry, servant leadership may alleviate emotional labor stress by understanding employees’ emotional needs and providing resource support, which should be positively associated with job satisfaction. Based on this, we propose the following hypothesis:

*H1*: Employee-perceived servant leadership is positively related to job satisfaction.

### 2.2 Psychological capital and its antecedents

Psychological capital (PsyCap) refers to an individual’s positive psychological state of development, characterized by (1) confidence (self-efficacy) to take on and put in the necessary effort to succeed at challenging tasks; (2) making a positive attribution (optimism) about succeeding now and in the future; (3) persevering toward goals and, when necessary, redirecting paths to goals (hope); and (4) sustaining and bouncing back from adversity (resilience) (Luthans et al., 2007). PsyCap is state-like rather than trait-like, meaning that it is malleable and can be developed through organizational interventions—a core tenet of positive organizational behavior (POB). Research indicates that employees with higher PsyCap levels are better equipped to cope with work-related stress, maintain positive emotions, and demonstrate greater adaptability when facing challenges (Avey et al., 2010). Leadership behavior is recognized as a critical situational factor influencing employees’ PsyCap.

Theoretically, this argument is consistent with both POB and COR. According to COR, supportive leadership behaviors function as external, situational resources that employees can draw upon; according to POB, such resources help employees develop and accumulate personal psychological resources. Specifically, servant leaders address employees’ needs, grant employees autonomy in their work, and provide development opportunities, which should strengthen employees’ self-efficacy and hope. Moreover, leaders’ support and understanding can reinforce employees’ resilience and optimism when they face challenges (Luthans et al., 2007; Hobfoll, 1989). Therefore, in the tourism industry, employees’ perception of servant leadership should facilitate the accumulation of their PsyCap. Based on this, the following hypothesis is proposed:

*H2*: Employee-perceived servant leadership is positively related to psychological capital.

### 2.3 The mediating role of psychological capital

Psychological capital has also been shown to be a key predictor of employees’ job satisfaction (Avey et al., 2010). Employees with higher PsyCap are more likely to experience positive emotions at work, to develop a positive perception of job meaning, and consequently to report higher job satisfaction. From the COR perspective, resource-rich individuals are less vulnerable to resource loss and better able to maintain positive evaluations of their work environment (Hobfoll, 1989). Moreover, PsyCap provides a theoretical bridge between leadership and job satisfaction: servant leadership supplies employees with situational resource support, while PsyCap serves as the mechanism through which employees internalize external resources into individual psychological advantages.

Based on the above analysis, this paper posits that PsyCap mediates the relationship between servant leadership and employee job satisfaction. Specifically, servant leadership should be positively related to employees’ PsyCap, which in turn should be positively related to their job satisfaction; in other words, servant leadership may promote job satisfaction partly by replenishing and building employees’ psychological resources. Accordingly, the following hypotheses are proposed:

*H3*: Psychological capital is positively related to job satisfaction.

*H4*: Psychological capital mediates the relationship between employee-perceived servant leadership and job satisfaction.

Consistent with Luthans et al. (2007), PsyCap is conceptualized as a higher-order construct comprising four components—hope, self-efficacy, resilience, and optimism. In addition to testing the overall logic of the model, and in line with recent research that examines PsyCap components separately, we test each of the four components as a distinct mediator. This component-level approach provides more fine-grained evidence on which psychological resource most strongly transmits the effect of servant leadership in the tourism context, without contradicting the higher-order conceptualization.

## 3 Research methods

### 3.1 Sample and data collection

The target population of this study comprised frontline employees of tourism enterprises (travel agencies, scenic spots, and hotels) in Yangtze River Delta cities of China, including Hefei, Shanghai, and Suzhou. Respondents were eligible to participate if they (1) were full-time employees, (2) worked in positions that involved direct, face-to-face service to tourists, and (3) had a direct supervisor at the time of the survey. A convenience sampling strategy was adopted.

Data were collected through a self-administered questionnaire in January 2026. With the assistance of human resource managers of the participating enterprises, questionnaires were distributed to frontline staff either in paper form or through an online survey platform. Before completing the questionnaire, each participant was informed of the study purpose, the anonymity of their responses, and their right to withdraw at any time; written informed consent was obtained. This study has obtained formal ethical approval, and relevant ethical documents are available upon request. To minimize common method bias, the survey was anonymous, and participants were assured that the data would be used solely for academic research and that there were no right or wrong answers. A total of 240 questionnaires were distributed, and 200 valid questionnaires were retained after excluding incomplete or patterned responses, yielding a valid response rate of 83.3% (200/240). The sample comprised 45.5% males and 54.5% females, predominantly aged 30–40 years, with an average tenure of approximately 9 years, indicating a representative sample structure.

### 3.2 Variable measurement

All measurement scales employed in this study were adapted from established international research, with minor wording adjustments to fit the tourism frontline context while preserving the original meaning of each item.

Servant leadership. Employee-perceived servant leadership was measured with six items adapted from the Servant Leadership Scale developed by Liden et al. (2008). As clarified in Section 2, the construct is conceptualized in line with Greenleaf’s (1970) servant leadership and is measured from the employees’ perspective. Several items were slightly reworded to fit frontline service scenarios.

Psychological capital. Psychological capital was measured with the 24-item Psychological Capital Questionnaire (PCQ-24) developed by Luthans et al. (2007), covering four dimensions—hope, self-efficacy, resilience, and optimism (six items each). Item wording was adapted where necessary to the tourism work context.Job satisfaction. Job satisfaction was measured with 16 items adapted from Zheng et al. (2015). All items were rated on a 5-point Likert scale ranging from 1 (“strongly disagree”) to 5 (“strongly agree”).

### 3.3 Data analysis methods

Data analysis was conducted with SPSS and AMOS software. The analytical strategy comprised five steps. First, data were screened and descriptive statistics were computed. Second, reliability was assessed with Cronbach’s alpha, and validity was examined through confirmatory factor analysis (CFA) using AMOS; convergent validity was evaluated with factor loadings, composite reliability (CR), and average variance extracted (AVE), and discriminant validity was assessed by comparing the square root of AVE with the inter-construct correlations. Third, Pearson correlations were computed among the study variables. Fourth, to test H1, hierarchical ordinary least squares (OLS) regression was performed with job satisfaction as the dependent variable; because age and years of service were strongly correlated, multicollinearity was examined through variance inflation factors (VIF) and tolerance values. Fifth, to test H2–H4, the Bootstrap method (5,000 resamples, 95% confidence intervals) was used to estimate the total, direct, and indirect effects of servant leadership on job satisfaction through each component of psychological capital (self-efficacy, hope, optimism, and resilience).

## 4 Empirical results

### 4.1 Statistical tools

Following data collection, invalid questionnaires (e.g., incomplete responses or clearly patterned answers) were removed, and descriptive statistics were computed using SPSS. Reliability and validity were then assessed as described in the Methods section. Because the study focuses on the direct and indirect (mediation) relationships among the variables, and because all constructs were measured with single-source self-report scales, hypotheses were tested with hierarchical OLS regression (for the direct effect of servant leadership on job satisfaction, H1) and Bootstrap mediation analysis (for the indirect effects through the four components of psychological capital, H2–H4).

The regression model was specifiedas:Y=β0+β1X+β2C+ε

Here, Y is the dependent variable (employee job satisfaction), X is the independent variable (employee-perceived servant leadership), C represents the control variables (gender, age, years of service, and marital status), and ε is the error term.In the mediation analysis, the mediating role of each component of psychological capital (self-efficacy, hope, optimism, and resilience) in the relationship between servant leadership and employee job satisfaction was explored. The Bootstrap method with 5,000 resamples was employed, and the total effect, direct effect, and indirect effect were estimated to determine whether each mediating path was significant.

The detailed results are presented below.

### 4.2 Descriptive statistical analysis

The demographic profile of the 200 respondents is summarized in Table 1. The sample was predominantly female (54.5%, *n* = 109), aged between 30 and 40 years (42.5% of the sample), married (67.5%, *n* = 134), and relatively experienced (49% had between 6 and 15 years of tenure). Table 1 reports the full distribution of each demographic category. These demographic variables were entered as control variables in the hypothesis tests; as reported below, none of them showed a statistically significant relationship with job satisfaction.

**Table 1:** Demographic profile of the respondents (*N* = 200).

Category	Subgroup	*n*	%
Gender	Male	91	45.5%
	Female	109	54.5%
Age	20–25 years	28	14.0%
	25–30 years	39	19.5%
	30–35 years	45	22.5%
	35–40 years	40	20.0%
	41–50 years	25	12.5%
	51 years and above	23	11.5%
Years of service	Less than 1 year	24	12.0%
	1–5 years	42	21.0%
	6–10 years	52	26.0%
	11–15 years	46	23.0%
	16 years and above	36	18.0%
Marital status	Unmarried	65	32.5%
	Married without children	34	17.0%
	Married with children	101	50.5%

### 4.3 Reliability and validity testing

### 4.4 Reliability analysis

Cronbach’s alpha was used to assess the internal consistency of the scales; values range from 0 to 1, with higher values indicating better internal consistency. The overall reliability of the questionnaire was excellent (*α* = 0.964), indicating that the measurement results are highly stable and suitable for the formal analyses.

Reliability analysis was conducted for all study constructs and dimensions, including servant leadership, the four components of psychological capital (hope, self-efficacy, resilience, and optimism), and job satisfaction. As shown in Table 2, all dimensions exhibited Cronbach’s alpha coefficients above the recommended threshold of 0.70, indicating strong internal consistency within each domain.

**Table 2:** Reliability analysis of the study variables.

Dimension	Number of items	Cronbach’s alpha
Servant leadership	6	0.844
Psychological capital – Hope	6	0.823
Psychological capital – Self-efficacy	6	0.866
Psychological capital – Resilience	6	0.779
Psychological capital – Optimism	6	0.803
Job satisfaction	16	0.941
Overall questionnaire	46	0.964
Service-oriented leadership	6	0.844
Employee job satisfaction	16	0.941
Efficacy	6	0.866
Hope	6	0.823
Optimistic	6	0.803
Tenacity	6	0.779

### 4.5 Validity analysis

Validity indicates the extent to which a measurement method effectively and accurately assesses the target variable. A validity test was conducted using SPSS: the Kaiser–Meyer–Olkin (KMO) value was 0.709 (>0.70), indicating that the data are suitable for factor analysis (0.70–0.80 is moderate, > 0.80 is good), and Bartlett’s test of sphericity was significant (χ^2^ = 1369.757, df = 528, *p* < 0.001), suggesting sufficient correlation among the items for factor analysis. Table 3 presents the KMO and Bartlett’s test results.To further evaluate the measurement model, confirmatory factor analysis (CFA) was conducted in AMOS. Standardized factor loadings ranged from 0.35 to 0.77, composite reliability (CR) values ranged from 0.79 to 0.94, and average variance extracted (AVE) values ranged from 0.40 to 0.52. All CR values exceeded 0.70, supporting internal consistency; however, four items (f4, xw1, rx2, and rx5) exhibited standardized loadings below 0.60 and four constructs (servant leadership, hope, resilience, and optimism) exhibited AVE values below 0.50, indicating that convergent validity was only partially supported. In addition, the square root of the AVE for each construct was not consistently greater than its correlations with the other constructs, so discriminant validity should be interpreted with caution. The overall fit of the measurement model was acceptable on most indices (χ^2^/df = 1.43, CFI = 0.92, TLI = 0.91, RMSEA = 0.047), although SRMR (0.11) slightly exceeded the recommended 0.08 threshold. The detailed CFA results are presented in Table 4.

**Table 3:** KMO and Bartlett’s test results.

Test	Value
Kaiser–Meyer–Olkin measure of sampling adequacy	0.709
Bartlett’s test of sphericity – Approximate chi-square	1369.757
Bartlett’s test of sphericity – df	528
Bartlett’s test of sphericity – Significance	0.000

**Table 4:** Confirmatory factor analysis results of the measurement model.

Construct	Standardized loadings (range)	CR	AVE
Servant leadership (6 items)	0.558–0.741	0.847	0.482
Psychological capital – Hope (6 items)	0.575–0.717	0.825	0.441
Psychological capital – Self-efficacy (6 items)	0.680–0.761	0.868	0.524
Psychological capital – Resilience (6 items)	0.352–0.744	0.790	0.397
Psychological capital – Optimism (6 items)	0.603–0.664	0.810	0.415
Job satisfaction (16 items)	0.639–0.770	0.943	0.508
Overall model fit: χ^2^/df	1.43		
CFI	0.92		
TLI	0.91		
RMSEA	0.047		
SRMR	0.112		

Although several AVE values fell slightly below the conventional cutoff of 0.50 and four items showed standardized loadings below 0.60 (with f4 exhibiting a particularly low loading), all composite reliability (CR) values exceeded 0.70, supporting acceptable internal consistency for each latent construct (Fornell and Larcker, 1981). The low-loading items were retained for two reasons. First, they were derived from widely used and well-validated classic scales (PCQ-24 and the Servant Leadership Scale of Liden et al. (2008); deleting them would alter the original scale structure and reduce comparability with prior tourism research. Second, a post-hoc item-deletion simulation indicated that removing these items produced only marginal improvements in AVE and model fit, without materially changing the pattern or significance of the hypothesized relationships. The SRMR value (0.11) was slightly higher than the recommended threshold of 0.08, suggesting that the measurement-model fit should be interpreted with caution. Given these caveats, the interpretation of the subsequent analyses focuses on the direction and statistical significance of the estimated effects rather than their absolute magnitudes.

### 4.6 Correlation analysis

Correlation analysis measures the strength and direction of the linear relationships between two variables, expressed as the Pearson correlation coefficient (r). Table 5 presents the correlations among job satisfaction, gender, age, years of service, marital status, and employee-perceived servant leadership. A significant positive correlation was observed between job satisfaction and employee-perceived servant leadership (*r* = 0.817, *p* < 0.01), indicating that higher levels of perceived servant leadership are associated with greater job satisfaction and providing preliminary support for H1. The correlations between the demographic variables and job satisfaction were weak and non-significant (gender: *r* = 0.080, *p* = 0.129; age: *r* = −0.005, *p* = 0.472; years of service: *r* = −0.040, *p* = 0.286; marital status: *r* = −0.074, *p* = 0.149), indicating that these demographic variables have minimal direct associations with job satisfaction.

**Table 5:** Correlations among the study variables (*N* = 200).

Variable	1	2	3	4	5	6
1. Job satisfaction	1.000	0.080	−0.005	−0.040	−0.074	0.817
2. Gender	0.080	1.000	−0.012	−0.041	−0.074	0.056
3. Age	−0.005	−0.012	1.000	0.936	0.773	−0.035
4. Years of service	−0.040	−0.041	0.936	1.000	0.769	−0.044
5. Marital status	−0.074	−0.074	0.773	0.769	1.000	−0.103
6. Servant leadership	0.817	0.056	−0.035	−0.044	−0.103	1.000

*N* = 200. *p* < 0.01 (one-tailed).

Age and years of service were highly correlated (*r* = 0.936, *p* < 0.01), which is logically consistent because age typically increases with years of service. Age and years of service were also positively correlated with marital status (*r* = 0.773 and 0.769, respectively, *p* < 0.01), indicating that employees’ marital status tends to change with age and tenure. Because of the very strong age–tenure correlation, multicollinearity was a concern when both variables are entered simultaneously as controls; VIF and tolerance statistics were therefore examined in the regression analyses (see below). The correlations between employee-perceived servant leadership and the demographic variables (gender, age, years of service, marital status) were low and non-significant, indicating that perceptions of servant leadership are largely independent of demographic factors and depend more on organizational management style and actual leadership behavior.

### 4.7 Regression analysis

Table 6 presents the hierarchical regression results. Model 1, which included only the control variables (marital status, gender, years of service, and age), yielded *R* = 0.151, *R*^2^ = 0.023, and adjusted *R*^2^ = 0.003, indicating that the control variables explained almost no variance in job satisfaction; the model was not statistically significant (*F* = 1.131, *p* = 0.343).

**Table 6:** Model summary of the hierarchical regression analyses.

Model	*R*	*R* ^2^	Adjusted *R*^2^	Std. error of the estimate	*R*^2^ change	*F* change	df1	df2	Sig. *F* change
1	0.151	0.023	0.003	0.566	0.023	1.131	4	195	0.343
2	0.822	0.675	0.667	0.327	0.652	389.257	1	194	0.000

Model 1 includes only the control variables (gender, age, years of service, and marital status); Model 2 adds employee-perceived servant leadership.

When employee-perceived servant leadership was added in Model 2, the model fit improved substantially: *R* increased to 0.822, *R*^2^ to 0.675, and adjusted *R*^2^ to 0.667, meaning that the model explained 67.5% of the variance in job satisfaction. The standard error of the estimate decreased from 0.566 to 0.327, the *R*^2^ change was 0.652, and the *F* change was significant (*F* = 389.257, *p* < 0.001), confirming the strong contribution of servant leadership.Overall, the control variables had minimal explanatory power for job satisfaction, whereas employee-perceived servant leadership substantially increased the explanatory power of the model. These findings provide preliminary support for H1.

The ANOVA results (Table 7) indicated that the regression model was statistically significant (*F* = 80.558, *p* < 0.001), suggesting that the set of predictors—marital status, gender, years of service, age, and servant leadership—significantly explained the variance in job satisfaction.

**Table 7:** ANOVA results.

Source	Sum of squares	df	Mean square	*F*	Sig.
Regression	43.184	5	8.637	80.558	0.000
Residual	20.799	194	0.107		
Total	63.983	199			

Dependent variable: job satisfaction. Predictors: gender, age, years of service, marital status, and employee-perceived servant leadership.

As shown in Table 8, in Model 1 none of the control variables was significantly related to job satisfaction: gender (*B* = 0.073, SE = 0.081, *t* = 0.907, *p* = 0.365), age (*B* = 0.112, SE = 0.076, *t* = 1.482, *p* = 0.140), years of service (*B* = −0.097, SE = 0.091, *t* = −1.063, *p* = 0.289), and marital status (*B* = −0.088, SE = 0.073, *t* = −1.211, *p* = 0.227).

**Table 8:** Regression coefficients.

Model	Variable	*B*	Std. error	Beta	*t*	Sig.
1	Constant	4.163	0.177		23.554	0.000
	Gender	0.073	0.081	0.065	0.907	0.365
	Age	0.112	0.076	0.307	1.482	0.140
	Years of service	−0.097	0.091	−0.218	−1.063	0.289
	Marital status	−0.088	0.073	−0.139	−1.211	0.227
2	Constant	0.933	0.193		4.834	0.000
	Gender	0.034	0.047	0.030	0.717	0.474
	Age	0.079	0.044	0.215	1.792	0.075
	Years of service	−0.094	0.053	−0.211	−1.775	0.077
	Marital status	0.005	0.042	0.008	0.120	0.905
	Servant leadership	0.756	0.038	0.815	19.730	0.000

*B* = unstandardized coefficient; Beta = standardized coefficient; dependent variable = job satisfaction.

In Model 2, employee-perceived servant leadership was strongly and significantly related to job satisfaction (*B* = 0.756, SE = 0.038, *β* = 0.815, *t* = 19.730, *p* < 0.001), demonstrating a strong positive association between this leadership style and job satisfaction.

In Model 2, gender and marital status remained non-significant, whereas the coefficients of age (*B* = 0.079, *t* = 1.792, *p* = 0.075) and years of service (*B* = −0.094, *t* = −1.775, *p* = 0.077) were marginally significant, suggesting weak associations with job satisfaction that did not reach statistical significance.In sum, individual background factors such as gender, age, marital status, and years of service were not significantly associated with job satisfaction, whereas employee-perceived servant leadership was strongly and positively associated with job satisfaction. This finding underscores the critical role of servant leadership in organizational management and its potential to promote employee wellbeing and job satisfaction; organizations and managers should therefore consider adopting this leadership style.

To test H1, employee-perceived servant leadership was entered as the independent variable and job satisfaction as the dependent variable, with gender, age, years of service, and marital status as control variables (Table 8). The results revealed a significant positive relationship between perceived servant leadership and job satisfaction (*B* = 0.756, *β* = 0.815, *p* < 0.001): on average, a one-point increase in perceived servant leadership was associated with a 0.756-point increase in job satisfaction. These results support H1. Among the control variables, age (*B* = 0.079, *p* = 0.075) and years of service (*B* = −0.094, *p* = 0.077) showed marginally significant coefficients, whereas gender (*B* = 0.034, *p* = 0.474) and marital status (*B* = 0.005, *p* = 0.905) were not significant.Multicollinearity diagnostics. Because age and years of service were highly correlated (*r* = 0.936), VIF and tolerance statistics were examined. Based on the correlation matrix (Table 5), the VIF values for age and years of service were approximately 8.1–8.5 (tolerance ≈ 0.12), exceeding the commonly recommended threshold of 5 and indicating substantial redundancy between the two variables. To further assess the robustness of this decision, an additional regression model was estimated in which only years of service, rather than both age and years of service, was retained as a control variable. In this robustness model, the effect of servant leadership on job satisfaction remained positive and statistically significant, and the magnitude of the coefficient changed only marginally; the complete results of the robustness analysis are available from the corresponding author upon request. Because the substantive conclusions were stable regardless of whether age was included, both control variables were retained in the main model, and this multicollinearity issue is acknowledged as a limitation in Section 5.4. Notably, the substantive conclusions were unaffected: the effect of servant leadership on job satisfaction remained highly significant after the controls were entered (*B* = 0.756, *p* < 0.001).

In conclusion, the results show that employee-perceived servant leadership is significantly positively associated with job satisfaction. Organizations should therefore actively encourage and cultivate such leadership behaviors to promote employee wellbeing. Furthermore, managers can implement tailored strategies—such as career development support and mental health counseling—according to employees’ age and tenure, thereby enhancing job satisfaction.

### 4.8 Mediation analysis of psychological capital

To further explore the mechanism through which employee-perceived servant leadership relates to job satisfaction, and to test the mediating roles of the four components of psychological capital, the Bootstrap method with 5,000 resamples and 95% confidence intervals was used. The results are presented in Tables 9–12. Psychological capital was conceptualized as a higher-order construct comprising four components—hope, self-efficacy, resilience, and optimism (Luthans et al., 2007). To provide more fine-grained evidence on which psychological resource most strongly transmits the effect of servant leadership in the tourism context, the four components were tested separately as mediators. This component-level approach complements, rather than contradicts, the higher-order conceptualization.

**Table 9:** Mediation results for self-efficacy.

Effect	Effect size	Boot SE	95% CI	Proportion of total effect
Total effect	0.756	0.038	[0.681, 0.832]	
Direct effect	0.560	0.052	[0.456, 0.663]	
Indirect effect	0.197	0.057	[0.094, 0.309]	26.06%

**Table 10:** Mediation results for hope.

Effect	Effect size	Boot SE	95% CI	Proportion of total effect
Total effect	0.756	0.038	[0.681, 0.832]	
Direct effect	0.611	0.045	[0.523, 0.700]	
Indirect effect	0.145	0.054	[0.051, 0.262]	19.18%

**Table 11:** Mediation results for optimism.

Effect	Effect size	Boot SE	95% CI	Proportion of total effect
Total effect	0.756	0.039	[0.681, 0.833]	
Direct effect	0.434	0.054	[0.328, 0.541]	
Indirect effect	0.323	0.074	[0.184, 0.466]	42.73%

**Table 12:** Mediation results for resilience.

Effect	Effect size	Boot SE	95% CI	Proportion of total effect
Total effect	0.761	0.038	[0.685, 0.836]	
Direct effect	0.595	0.045	[0.505, 0.684]	
Indirect effect	0.166	0.049	[0.077, 0.260]	21.81%

In the mediation analysis of self-efficacy (Table 9), the total effect was 0.756, the direct effect was 0.560, and the indirect (mediating) effect was 0.197. The 95% confidence intervals of the total effect ([0.681, 0.832]), the direct effect ([0.456, 0.663]), and the indirect effect ([0.094, 0.309]) did not include zero, indicating that self-efficacy significantly mediated the relationship between employee-perceived servant leadership and job satisfaction. Thus, H4 was supported for self-efficacy, with the indirect effect accounting for 26.06% of the total effect.In the mediation analysis of hope (Table 10), the total effect was 0.756, the direct effect was 0.611, and the indirect effect was 0.145. The 95% confidence intervals of the total effect ([0.681, 0.832]), the direct effect ([0.523, 0.700]), and the indirect effect ([0.051, 0.262]) did not include zero, indicating that hope significantly mediated the relationship between employee-perceived servant leadership and job satisfaction. Thus, H4 was supported for hope, with the indirect effect accounting for 19.18% of the total effect.

In the mediation analysis of optimism (Table 11), the total effect was 0.756, the direct effect was 0.434, and the indirect effect was 0.323. The 95% confidence intervals of the total effect ([0.681, 0.833]), the direct effect ([0.328, 0.541]), and the indirect effect ([0.184, 0.466]) did not include zero, indicating that optimism significantly mediated the relationship between employee-perceived servant leadership and job satisfaction. Thus, H4 was supported for optimism, with the indirect effect accounting for 42.73% of the total effect.

In the mediation analysis of resilience (Table 12), the total effect was 0.761, the direct effect was 0.595, and the indirect effect was 0.166. The 95% confidence intervals of the total effect ([0.685, 0.836]), the direct effect ([0.505, 0.684]), and the indirect effect ([0.077, 0.260]) did not include zero, indicating that resilience significantly mediated the relationship between employee-perceived servant leadership and job satisfaction. Thus, H4 was supported for resilience, with the indirect effect accounting for 21.81% of the total effect.

In summary, the correlation analysis revealed a significant positive correlation between employee-perceived servant leadership and job satisfaction (*r* = 0.817, *p* < 0.01), providing preliminary support for H1; the mediation analyses further revealed significant relationships among servant leadership, each component of psychological capital, and job satisfaction. Confirmatory factor analysis of the measurement model showed that composite reliability (CR) exceeded 0.70 for all constructs, whereas four items exhibited standardized loadings below 0.60 and four constructs exhibited AVE values below 0.50, indicating that convergent validity was only partially supported; discriminant validity and the overall model fit should therefore be interpreted with caution (see Table 4). The regression and Bootstrap analyses showed that employee-perceived servant leadership was significantly positively associated with job satisfaction (H1 supported); the significant indirect paths implied that servant leadership was significantly positively associated with each component of psychological capital (consistent with H2) and that each component was significantly positively associated with job satisfaction (consistent with H3); and psychological capital partially mediated the relationship between servant leadership and job satisfaction (H4 supported). It should be noted that these dimension-specific indirect effects do not directly constitute full support for H2 and H3, which were formulated for the overall higher-order psychological capital construct; rather, they indicate that both constituent paths within each dimension-level mediation are statistically non-zero.

The Bootstrap mediation results further show that the indirect effects of servant leadership on job satisfaction through the four components of psychological capital were all significant (the 95% confidence intervals did not include zero), indicating that psychological capital plays a partial mediating role between servant leadership and employee job satisfaction. Therefore, H4 is supported.

## 5 Discussion and conclusion

### 5.1 Discussion of research findings

The data analysis reveals that employee-perceived servant leadership is significantly positively associated with job satisfaction among tourism employees, further supporting its positive role in service-intensive sectors. Unlike traditional task-oriented leadership, servant leadership creates a supportive work environment by addressing employee needs and respecting individual values, thereby strengthening positive emotional experiences and, in turn, job satisfaction.

Further analysis reveals that employees’ perceived servant leadership is significantly positively associated with their psychological capital, indicating that leaders’ servant behaviors relate not only to employees’ overt attitudes but also to their internal psychological resource structures. This finding provides empirical evidence from the perspective of positive organizational behavior, supplementing the understanding of how servant leadership relates to employees’ psychological states.

Meanwhile, psychological capital was significantly positively associated with employees’ job satisfaction and partially mediated the relationship between servant leadership and job satisfaction. This finding indicates that servant leadership relates to job satisfaction not only directly but also indirectly by building employees’ positive psychological resources (psychological capital), which help them cope with work-related stress and emotional labor and ultimately lead to higher job satisfaction.

### 5.2 Theoretical contributions

This study makes three theoretical contributions. First, it deepens research on servant leadership from the perspective of employee perception (addressing Gap 1). While existing studies predominantly focus on leadership behaviors themselves, this paper emphasizes the critical role of employees’ subjective perceptions of leadership behaviors in shaping psychological and attitudinal outcomes, thereby expanding the research perspective on the mechanisms of servant leadership.

Second, this paper introduces psychological capital as a mediator to reveal the internal psychological mechanism through which servant leadership relates to employees’ job satisfaction (addressing Gap 2). By empirically testing the “servant leadership—psychological capital—job satisfaction” pathway, it addresses the inadequacy of previous research regarding leadership-behavior mechanisms. Going beyond a composite PsyCap score, the component-level analyses further identify which psychological resource is the strongest transmitter in the tourism context (optimism, accounting for 42.7% of the total effect), a fine-grained insight that studies modeling PsyCap only as a composite cannot provide.

Third, this study focuses on tourism industry employees, enriching the contextual evidence on servant leadership and employee wellbeing (addressing Gap 3). The tourism industry is characterized by high emotional labor and high service intensity, and the findings help validate the applicability and explanatory power of the relevant theories in service-intensive industries.

### 5.3 Practical implications

The findings of this study provide important practical implications for human resource management in tourism enterprises. First, tourism enterprises should prioritize cultivating servant leadership by selecting and training managers with a caring awareness and service orientation, thereby creating a supportive organizational atmosphere and enhancing employees’ job satisfaction.

Second, enterprises should regard employees’ psychological capital as an important part of sustainable human resource management and help employees accumulate positive psychological resources through systematic training, psychological counseling, and career development support, so as to enhance their ability to cope with high-intensity service work.

Third, tourism enterprises should incorporate employees’ job satisfaction into their management objectives while maintaining attention to service performance, so as to avoid relying solely on performance appraisal and neglecting employees’ psychological needs; this can achieve the dual improvement of employees’ job satisfaction and organizational performance.

### 5.4 Limitations and future research directions

Although this study has yielded informative findings, several limitations should be acknowledged. First, the cross-sectional design means that causal relationships among the variables cannot be rigorously established; the language used throughout this paper (e.g., “associated with” rather than “causes”) reflects this limitation. Future research could employ longitudinal or multi-wave designs to examine the proposed relationships over time. Second, the sample was primarily drawn from the tourism industry in one region, and the applicability of the findings to other sectors and regions requires further validation. Third, because all variables were measured with self-report questionnaires at a single point in time, common method bias cannot be fully ruled out; future studies could collect data from multiple sources or at multiple time points. Fourth, the very strong correlation between age and years of service (*r* = 0.936) indicates that these two control variables carry largely redundant information; future studies should select control variables more carefully.

In addition, future research could introduce situational variables or individual trait variables—such as work–family interface factors (Greenhaus and Beutell, 1985)—to further explore the boundary conditions of servant leadership and employee wellbeing.

At the same time, future research could model psychological capital as a higher-order construct with larger samples, or examine whether its four components operate jointly rather than separately, to deepen the understanding of its mechanism of action.

## Data Availability

The raw data supporting the conclusions of this article will be made available by the authors, without undue reservation.
